# Functional characterization of cinnamyl alcohol dehydrogenase and caffeic acid *O*-methyltransferase in *Brachypodium distachyon*

**DOI:** 10.1186/1472-6750-13-61

**Published:** 2013-07-31

**Authors:** Gina M Trabucco, Dominick A Matos, Scott J Lee, Aaron J Saathoff, Henry D Priest, Todd C Mockler, Gautam Sarath, Samuel P Hazen

**Affiliations:** 1 Biology Department, University of Massachusetts 221 Morrill Science Center III, Amherst, MA 01003, USA; 2 Molecular and Cellular Biology Graduate Program, University of Massachusetts, Amherst, MA, USA; 3 Plant Biology Graduate Program, University of Massachusetts, Amherst, MA, USA; 4 USDA-ARS, Grain, Forage, and Bioenergy Research Unit, University of Nebraska-Lincoln, Lincoln, NE, USA; 5 The Donald Danforth Plant Science Center, St. Louis, MO, USA

## Abstract

**Background:**

Lignin is a significant barrier in the conversion of plant biomass to bioethanol. Cinnamyl alcohol dehydrogenase (CAD) and caffeic acid *O*-methyltransferase (COMT) catalyze key steps in the pathway of lignin monomer biosynthesis. Brown midrib mutants in *Zea mays* and *Sorghum bicolor* with impaired CAD or COMT activity have attracted considerable agronomic interest for their altered lignin composition and improved digestibility. Here, we identified and functionally characterized candidate genes encoding CAD and COMT enzymes in the grass model species *Brachypodium distachyon* with the aim of improving crops for efficient biofuel production.

**Results:**

We developed transgenic plants overexpressing artificial microRNA designed to silence *BdCAD1* or *BdCOMT4*. Both transgenes caused altered flowering time and increased stem count and weight. Downregulation of *BdCAD1* caused a leaf brown midrib phenotype, the first time this phenotype has been observed in a C_3_ plant. While acetyl bromide soluble lignin measurements were equivalent in *BdCAD1* downregulated and control plants, histochemical staining and thioacidolysis indicated a decrease in lignin syringyl units and reduced syringyl/guaiacyl ratio in the transgenic plants. *BdCOMT4* downregulated plants exhibited a reduction in total lignin content and decreased Maule staining of syringyl units in stem. Ethanol yield by microbial fermentation was enhanced in *amiR*-*cad1*-*8* plants.

**Conclusion:**

These results have elucidated two key genes in the lignin biosynthetic pathway in *B*. *distachyon* that, when perturbed, may result in greater stem biomass yield and bioconversion efficiency.

## Background

Plant biomass offers a sustainable, low-carbon-emitting source of biofuel feedstock to potentially alleviate both environmental and economic disadvantages of fossil fuel usage [1]. Fossil fuel combustion has resulted in elevated atmospheric CO_2_ levels that continue to rise, threatening air quality, wildlife habitat, and human health [2,3]. A viable, cost-effective alternative is to replace and/or blend gasoline and diesel fuels with biofuel. The efficiency of existing approaches to generating biofuels can be improved through research into plant feedstock attributes, namely biomass yield and recalcitrance to conversion [4].

Plant cell walls are mostly comprised of cellulose, hemicellulose, and lignin. The composition and interaction among these three constituents largely dictate the amenability of a plant feedstock for conversion to simple sugars and then to biofuels. Cellulose and hemicellulose are important raw materials, for they are an abundant source of monomeric sugars that can be hydrolyzed by enzymes and fermented by microbes, while lignin cannot [5]. Due to the recalcitrance of plant cell walls, a strong acid, heat, or other chemical pretreatment is required to first break up cell wall fibers and access the polysaccharides for enzyme treatment. Lignin in particular can impede the pretreatment process by adhering to the hydrolytic enzymes used to saccharify cellulose and other polysaccharides, affecting their ability to work efficiently. Additionally, some lignin degradation products can inhibit subsequent fermentation steps by releasing aromatic compounds that inhibit enzymes used for converting sugars to ethanol [6]. Saccharification of *Medicago sativa* expressing antisense transcripts designed to silence lignin biosynthesis genes showed an inverse relationship between lignin content and sugar yield, revealing that lignin is indeed a significant obstacle to obtaining high yields of cell wall sugars [7]. Consequently, there has been strong interest in the engineering of bioenergy crops, namely grasses, that are more amenable to feedstock conversion, notably by the manipulation of lignin biosynthesis, in order to improve efficiency of the pretreatment process and obtain maximum fuel yield [8].

Lignin is a complex phenolic polymer, and despite its recalcitrant properties, it is important in providing structural support, hydrophobicity, and protection against pathogens [9]. It is comprised of *p*-hydroxyphenyl (H), guaiacyl (G), and syringyl (S) units derived from the monolignol precursors *p*-coumaryl, coniferyl, and sinapyl alcohols, respectively. A series of ten enzymes is involved in the phenylpropanoid pathway for monolignol biosynthesis [9]. The eudicot *Arabidopsis thaliana* has served as a valuable tool to understand lignin biosynthesis [9]. However, there are major plant cell wall structural differences between eudicots and grasses including the type and abundance of lignocellulosic components, pectins, and proteins and the linkages between them [10,11].

Cinnamyl-alcohol dehydrogenase (CAD) functions in one of the final steps of monolignol biosynthesis that catalyzes the reduction of cinnamyl aldehyde to cinnamyl alcohol prior to polymerization into the lignin polymer. The highly conserved Rossmann fold NAD(P)H/NAD(P)+ binding domain found in CAD monolignol-synthesizing proteins indicates the use of NADPH as a cofactor in the reduction reaction [12]. *CAD* tends to exist in multi-gene families with one gene being primarily responsible for lignin biosynthesis. The effect of *CAD* downregulation has been studied using a transgenic approach in several eudicot species including *Nicotiana tabacum*[13-16], *M. sativa*[17], *Populus sp.*[18-20] and *Eucalyptus camaldulensis*[21]. Reports of lignin modification by downregulation of *CAD* in grasses are limited to *Panicum virgatum*[22,23], *Zea mays*[24], and *Festuca arundinacea*[25]. In general, the transgenic downregulation of *CAD* does not affect total lignin content; instead, the inhibition of monolignol biosynthesis leads to changes in lignin composition, such as incorporation of accumulated aldehyde precursors or novel units into the lignin polymer, changes that make biomass more digestible [26].

Caffeic acid *O*-methyltransferase (COMT) is an *O*-methyltransferase that tends to be broad in substrate affinity and can potentially act in various branches of the phenylpropanoid pathway. The highly conserved S-adenosyl methionine (SAM) binding domain in COMT proteins indicates the use of SAM as the methyl group donor to the hydroxyl group of a methyl acceptor molecule [27]. COMT is involved in the methylation of caffeic acid to ferulic acid, which is then hydroxylated at position five by ferulate-5-hydroxylase. The subsequent methylation by COMT at this position yields sinapic acid. Similarly, COMT catalyzes the 3-*O*-methylation of caffeal- and coniferal- aldehyde/alcohol precursors to G and S lignin [28,29]. In *Brachypodium distachyon*, COMT has high affinity for a variety of substrates including flavonoid compounds, with the greatest activity with caffeic acid and caffealdehyde [30]. Transgenic downregulation of *COMT* has been reported in eudicots including *M. sativa*[31], *N. tabacum*[32,33], and *Populus sp.*[18,34,35]. Reports of *COMT*-downregulated transgenic grasses are more limited, including *P. virgatum*[36], *Z. mays*[37], *F.arundinacea*[38] and *Lolium perenne*[39]. Perturbation of the COMT enzyme often results in the inhibition of S lignin formation and consequently the accumulation of 5-OH coniferyl alcohol that could not be synthesized into S lignin is instead converted into a G lignin monomer, resulting in incorporation of a novel 5-hydroxyguaiacyl lignin unit into the lignin polymer [33,40]. Accordingly, *COMT* transgenics with altered lignin composition tend to be more digestible [26].

In the early 1920’s, *brown midrib* (*bmr*) mutants were identified in *Z. mays* as displaying a brownish-red to tan pigmentation of the leaf midrib associated with reduced lignin content or altered lignin composition [41]. A similar discoloration was observed in lignified tissues in the stalk. Changes in development are also apparent, as exemplified by variation in flowering time [42]. While the *bmr* mutants tend to have improved digestibility, various unfavorable traits including decreased grain and stem yield, increased lodging, and increased disease susceptibility sometimes develop [40,43,44]. Mutants have been isolated in the C_4_ grasses *Z. mays*, *S. bicolor*, and *Pennisetum glaucum*. Four *bmr* loci in *Z. mays*, *bm1*, *bm2*, *bm3* and *bm4*, represent spontaneous mutants that were first isolated almost a century ago; an additional locus, *bm5*, was identified later [40]. In *S. bicolor*, four brown midrib loci, *bmr2*, *bmr6*, *bmr12*, and *bmr19*, were isolated from mutagenized populations [45,46]. Three brown midrib mutants have been isolated in *P. glaucum*, although they have not been as well characterized [47]. In *Z. mays* and *S. bicolor*, the *bmr* phenotype is associated with orthologous loci that encompass *CAD* and *COMT* genes. The *bm1* mutation affects expression of *ZmCAD2* and five alleles of the orthologous *bmr6* have been characterized in *S. bicolor*[48-51]. The *bm3* locus in *Z. mays* and *bmr12* in *S. bicolor* correspond to orthologous *COMT* genes [52,53]. The *bmr* mutants have the potential to act as viable bioenergy crops, as the visual phenotype seems to be an effective marker for impaired lignin biosynthesis associated with improved digestibility. A more complete understanding of the genes responsible for the phenotype will help provide novel breeding strategies and expand the resources of conversion-efficient plants.

Grasses are a key source of grain and forage and have recently gained in importance as feedstocks for the biofuel industry. Large perennial bioenergy crops such as *P. virgatum* and *Miscanthus sp.* have a relatively long generation time, complex genomes, and tall stature that make these species difficult research subjects. In order to better understand grasses, the small annual grass, *B. distachyon*, was used here as a research model from which we can translate results to the improvement of crops for efficient biofuel production [54]. It has many attributes of a model system including a small diploid and sequenced genome, rapid generation time, short stature, and it is easily transformable [54]. In this study, we used artificial microRNAs to disrupt the function of candidate *CAD* or *COMT* genes, with the objective of characterizing their role in lignin biosynthesis in *B. distachyon*.

## Results

### Characterization and phylogenetic analysis of *BdCAD* and *BdCOMT* gene families

**Figure 1:**
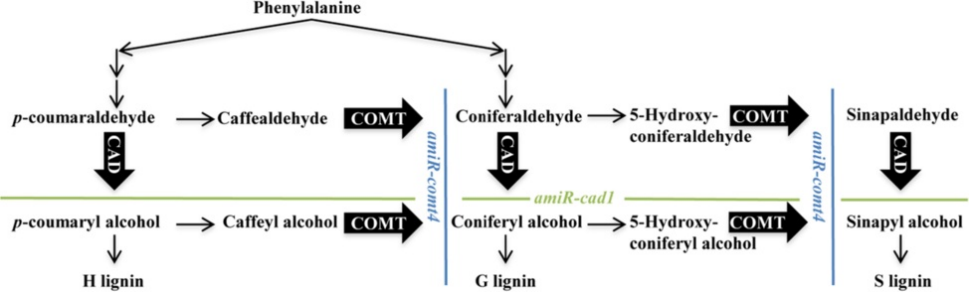
**A simplified diagram of the phenylpropanoid pathway for lignin biosynthesis.** Cinnamyl alcohol dehydrogenase (CAD) converts the cinnamyl aldehydes (*p*-coumaraldehyde, coniferaldehyde and sinapaldehyde) to alcohols, the monomeric precursors of H, G, and S lignin. Caffeic acid *O*- methyltransferase (COMT) catalyzes the methylation of alcohol/aldehyde precursors to S and G lignin. *amiR-cad1* impairs CAD activity; *amiR-comt4* impairs COMT activity.

**Figure 2:**
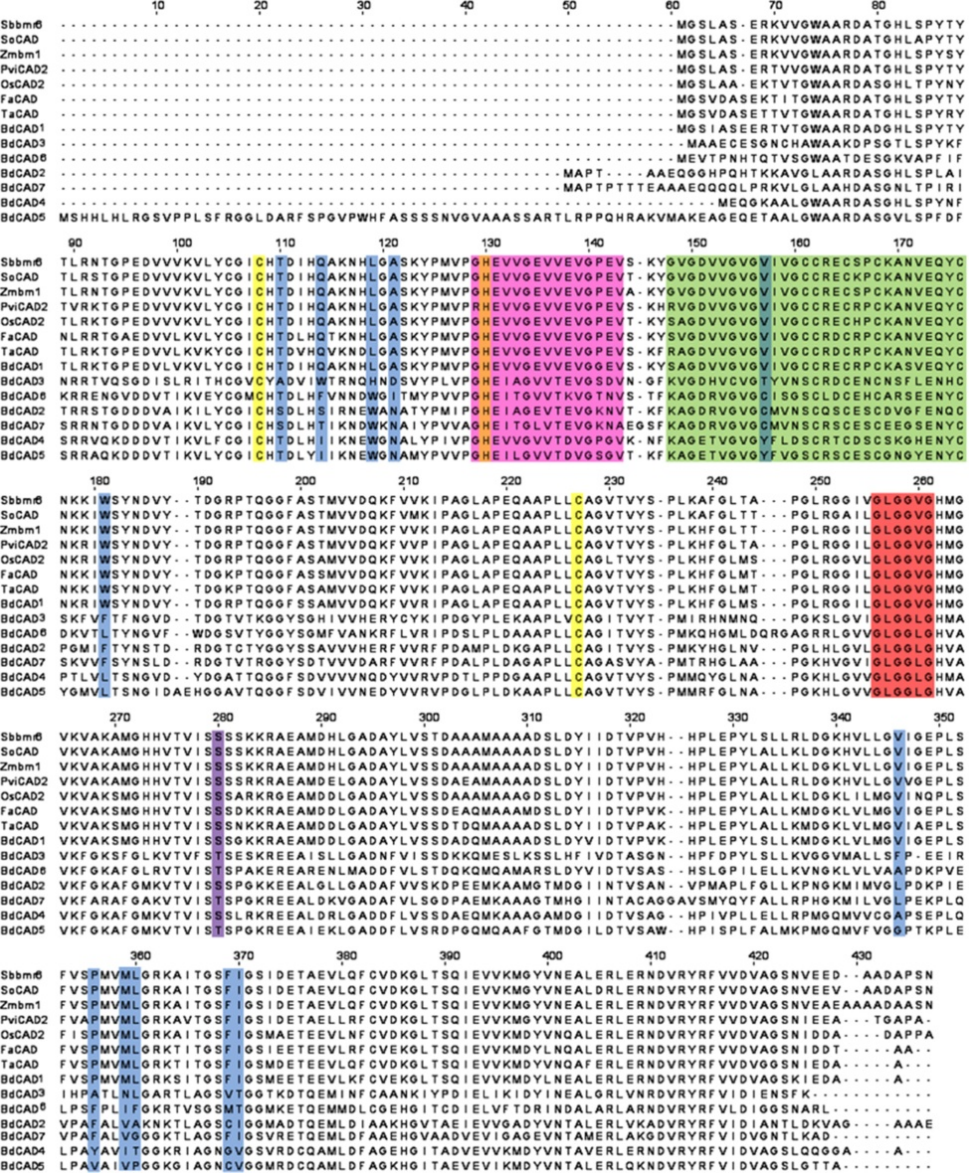
**Sequence alignment of the CAD family of *Brachypodium distachyon *and other species.** Amino acid sequence comparison of seven *B. distachyon* CAD proteins with other functionally characterized CAD proteins in *Sorghum bicolor* (Sbbmr6), *Saccharum officinarum* (SoCAD), *Zea mays* (Zmbm1), *Panicum virgatum (*PviCAD2), *Oryza sativa* (OsCAD2), *Festuca arundinacea* (FaCAD), and *Triticum aestivum* (TaCAD). The Zn-1 binding domain motif is labeled in pink and Zn-1 catalytic residues are labeled in yellow. The NADP(H) cosubstrate-binding domain motif is labeled in red and the Zn-2 structural motif is labeled in green. Substrate-binding residues are labeled in blue, and the Ser212 specific NADP(H) binding residue is labeled in purple.

Given their importance to monolignol synthesis in other plants, *CAD* and *COMT* genes with a potential role in secondary cell wall lignification were identified and perturbed in order to evaluate their role in *B. distachyon* (Figure 1). Candidate genes were selected based on sequence homology to previously characterized genes. Seven putative BdCADs were identified by BLAST search of the *B. distachyon* genome with the *Oryza sativa* CAD protein family [55]. The BdCAD family consists of Bradi3g06480 (BdCAD1), Bradi3g17920 (BdCAD2), Bradi3g22980 (BdCAD3), Bradi4g29770 (BdCAD4), Bradi4g29780 (BdCAD5), Bradi5g04130 (BdCAD6), and Bradi5g21550 (BdCAD7); numbered sequentially as they appear in the genome. Multiple amino acid sequence alignment was performed with the seven candidate *B. distachyon* CAD family members along with *S. bicolor* (Sbbmr6), *Saccharum officinarum* (SoCAD), *Z. mays* (Zmbm1), *P. virgatum* (PviCAD), *O. sativa* (OsCAD2), *F. arundinacea* (FaCAD), and *Triticum aestivum* (TaCAD) CAD proteins. Alignment results indicate a high degree of similarity in conserved domains and binding residues characteristic of alcohol dehydrogenases, especially in BdCAD1 (Figure 2). All seven CAD family members in *B. distachyon* contain the Zn-1 binding domain motif GHE(X)_2_G(X)_5_G(X)_2_V and the conserved Zn-1 catalytic residues C47, H69, and C163. The sequence of the Zn-1 binding motif is most highly conserved in BdCAD1, with 99.3% homology to the motif in the other aforementioned grass species CAD proteins. A glycine-rich repeat GXG(X)_2_G, involved in NADP(H) cosubstrate-binding, is conserved amongst all CAD proteins. The consensus sequence GD(X)_9,10_C(X)_2_C(X)_2_C(X)_7_C for binding the Zn-2 metal ion is preserved in the BdCADs. Additionally, twelve amino acids have been identified as substrate-binding residues in the *bona fide* CADs of various plant species [56]. Of the seven CADs in *B. distachyon*, BdCAD1 contains ten of these twelve conserved residues, while the other family members are more variable at these positions. Interestingly, only BdCAD1 contains both active substrate-binding residues, W119 and F298, which determine specificity for aromatic alcohols, and the conserved S212 residue that determines NADP(H) binding at that position, as seen in OsCAD2 in rice [55]. Pairwise sequence alignments with the BdCAD1 protein revealed high percent identity to *F. arundinacea* (89.7%), *T. aestivum* (89.4%), *O. sativa* (89.2%), *P. virgatum* (87.5%), *Z. mays* (88.3%), *S. officinarum* (86.9%), and *S. bicolor* (86.9%). Based on amino acid sequence, it appears that BdCAD1 (Bradi3g06480) contains the conserved functional and structural features of a medium chain dehydrogenase/reductase specific to enzymes involved in lignin biosynthesis in secondary cell walls.

**Figure 3:**
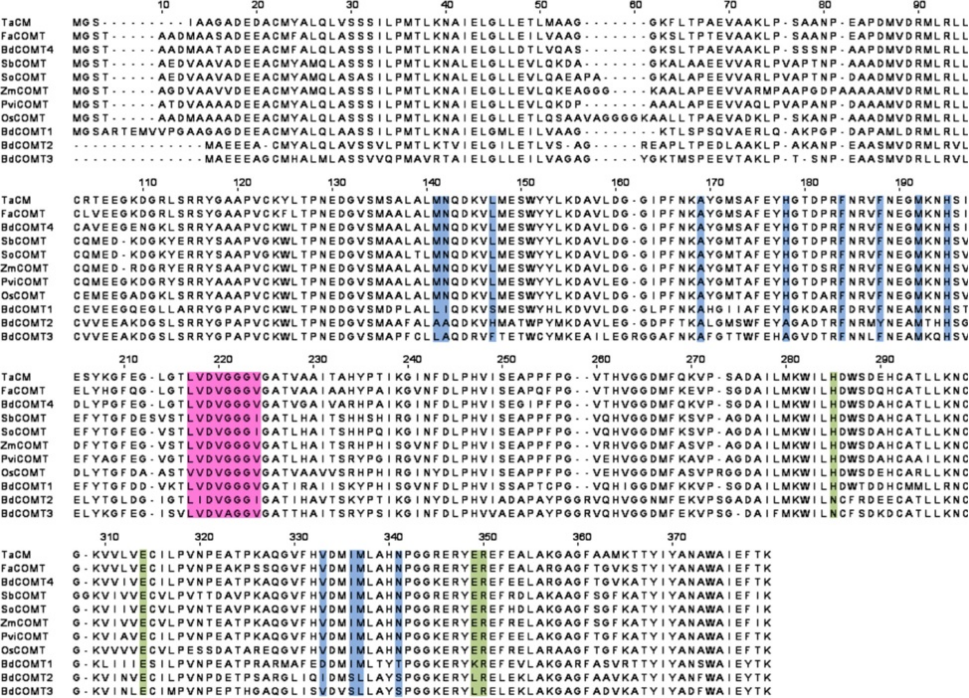
**Sequence alignment of the COMT family of *Brachypodium distachyon *and other species.** Amino acid sequence comparison of the four COMT proteins in *B. distachyon* with functionally characterized COMT proteins in *Triticum aestivum* (TaCM)*, Festuca arundinacea* (FaCOMT)*, Sorghum bicolor* (Sbbmr12), *Zea mays* (Zmbm3), *Panicum virgatum* (PviCOMT), and *Oryza sativa* (OsCOMT)*.* The conserved motif for the SAM binding domain is labeled in pink, catalytic residues are labeled in green, and active site substrate-binding residues are labeled in blue.

Four COMTs were identified in *B. distachyon*, Bradi1g14870 (BdCOMT1), Bradi2g02380 (BdCOMT2), Bradi2g02390 (BdCOMT3), and Bradi3g16530 (BdCOMT4). Multiple amino acid sequence alignment was then performed with the four derived *B. distachyon* COMT family members and with known COMTs in *T. aestivum* (TaCM), *F. arundinacea* (FaCOMT), *S. bicolor* (Sbbmr12), *S. officinarum* (SoCOMT), *Z. mays* (Zmbm3), and *P. virgatum* (PviCOMT). Sequence comparison with the functionally characterized COMT proteins from various plants showed a high degree of similarity at the amino acid level (Figure 3). The S-adenosyl-L-methionine (SAM) binding domain, LVDVGGGxG, a signature of *O*-methyltransferases, is conserved in BdCOMT1, BdCOMT3, and BdCOMT4; however, one residue is inconsistent at position 193 in BdCOMT2, where isoleucine replaces valine in the sequence motif. Catalytic residues E310, E342, and R343 are conserved in all *B. distachyon* COMT family members and all other characterized COMT lignin proteins. Interestingly, catalytic residue H281 found in *P. virgatum*, *F. arundinacea*, *S. bicolor*, and *Z. mays*, is conserved only in BdCOMT1 and BdCOMT4. Of the four BdCOMT family members, only BdCOMT4 has all substrate-binding and positioning residues M130, N131, L136, A162, H166, F176, M180, H183, I319, M320, N324 conserved in the known COMTs specific to the synthesis of secondary cell wall lignin. Pairwise sequence alignments with BdCOMT4 revealed high percent identity to *F. arundinacea* (89.2%), *T. aestivum* (87.4%), *O. sativa* (83.4%), *P. virgatum* (83.4%), *S. officinarum* (80.2%), *Z. mays* (80.0%), and *S. bicolor* (78.8%). The prevalence of common signatures in the amino acid sequence supports the idea that BdCOMT4 is a COMT ortholog functioning as a SAM-dependent *O*-methyltransferase in *B. disatchyon*.

**Figure 4:**
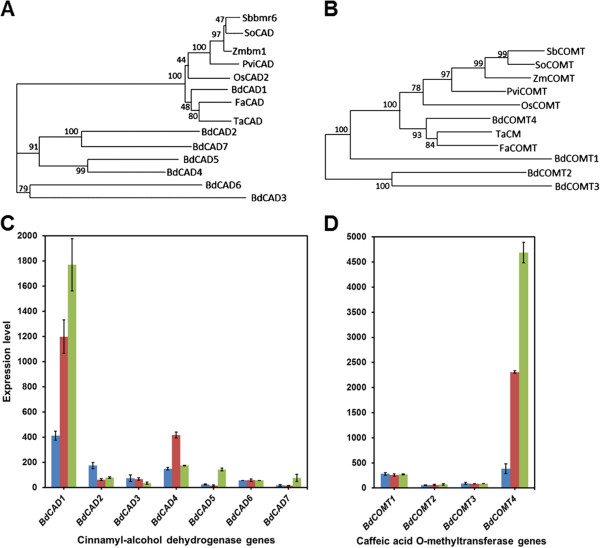
**Phylogenetic analysis and expression patterns of CAD and COMT. (A,B)** Unrooted phylogenetic tree of *B. distachyon* CAD and COMT families and characterized proteins from other grasses. A multiple amino acid sequence alignment was performed with ClustalW and the phenogram was constructed with MEGA neighbor-joining method with 1000 bootstrap permutations. **(C,D)** Anatomical gene expression in leaf (blue), root (red), and stem (green) of *CAD* and *COMT* families in *B. distachyon*.

Evidence for the role of BdCAD1 and BdCOMT4 as functional lignin biosynthesis proteins was further enhanced by phylogenetic analysis. An unrooted neighbor-joining tree with 1000 bootstrap permutations was generated from the deduced multiple amino acid sequence alignments of the aforementioned CAD and COMT proteins. The phenogram established a single clade in which the characterized CAD proteins known to be involved in monolignol biosynthesis in monocots were clustered with one protein from our candidate *B. distachyon* family, BdCAD1 (Figure 4A). Within that clade, BdCAD1 clustered with *F. arundinacea*, *T. aestivum* and *O. sativa* proteins. A second, closely related group consisted of CADs in *P. virgatum*, *Z. mays*, *S. bicolor*, and *S. officinarum*. The remaining seven CAD family members in *B. distachyon* did not fall into any such existing clades. A similar trend was observed in the phylogenetic analysis of the COMT sequences isolated from the previously mentioned species. A common clade formed amongst the known COMT lignin proteins and included one COMT from the *B. distachyon* family, BdCOMT4 (Figure 4B). Within the clade, BdCOMT4 clustered with *F. arundinacea* and *T. aestivum* proteins, while a closely related group consisted of *P. virgatum*, *Z. mays*, *S. bicolor*, and *S. officinarum* proteins. In contrast, the other three BdCOMTs did not show a similar relationship to known COMT enzymes. The relationships among the CAD and COMT sequences specifically portray *BdCAD1* and *BdCOMT4* as unique from any other candidates in the corresponding multi-gene families in *B. distachyon.*

### *BdCAD1* and *BdCOMT4* are highly expressed in developing stem

Anatomical expression data were also considered in evaluating the multi-gene *BdCAD* and *BdCOMT* families for a role in lignification. We used microarray data to analyze gene expression of each member of the family in developing stems, roots, and leaves. Lignin biosynthesis genes are expected to be highly expressed in stems, where secondary cell walls are prevalent and lignification occurs, while remaining at relatively low levels in roots and especially leaves. Of the seven CAD genes identified in *B. distachyon*, *BdCAD1* expression was greatest in stem tissue, exhibiting ten-fold higher transcript level than any of the other seven *BdCAD* genes (Figure 4C). Expression of *BdCOMT4* was also greatest in the stem and was eighteen-fold greater than the expression of the other three *BdCOMT* genes (Figure 4D). As might be expected, both *BdCAD1* and *BdCOMT4* were expressed at a slightly lower level in roots, where lignin is also present, and at a significantly lower level in leaves, where there is not much lignin at all.

### *BdCAD1* and *BdCOMT4* transgenic plants

**Figure 5:**
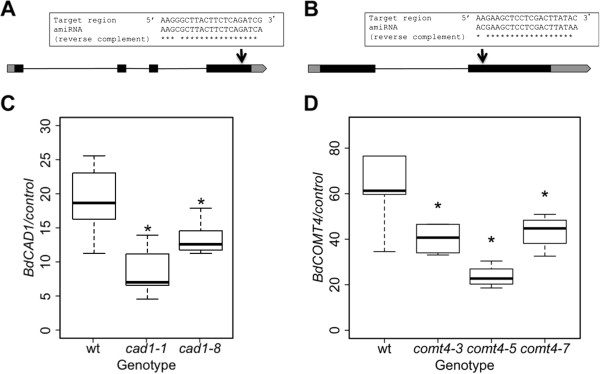
**Design of artificial microRNA transgene constructs and relative expression of *BdCAD1 *and *BdCOMT4 *in transgenic plants.** Artificial microRNA (amiRNA) constructs were designed to target and silence the **(A)***BdCAD1* and **(B)***BdCOMT4* transcripts. Exons are indicated by solid black boxes, untranslated regions by grey boxes, and intron by thin, black lines. The genes are drawn to scale. Arrows indicate the artificial microRNA target regions. RNA was prepared from stems of developmentally equivalent transgenic and empty vector control plants and subjected to quantitative real-time PCR to assay for target gene expression. Relative expression of **(C)***BdCAD1* and **(D)***BdCOMT4* with control housekeeping gene Bradi5g25870. The boxes show interquartile range, the whiskers show the outer quartile edge, and the black line represents the median of each distribution. Open circles represent outliers, when present. * Denotes significance at the 5% level.

In order to functionally characterize *BdCAD1* and *BdCOMT4*, reduction-of-function mutants were developed and assayed for changes in secondary cell wall composition. Highly specific artificial microRNA (amiRNA) constructs were designed to target and silence *BdCAD1* or *BdCOMT4* (Figure 5A, B). The WMD Version 3 web-based tool was used to design amiRNA sequences for the transgenes. The *BdCAD1* transcript was targeted by a 21-mer sequence at nucleotides 950–970 in the fourth exon (Figure 5A). The *BdCOMT4* transcript was targeted by a 21-mer sequence at nucleotides 556–576 in the second exon (Figure 5B). Two transformation events targeting *BdCAD1* (*amiR-cad1-1* and *amiR-cad1-8*) and three transformation events targeting *BdCOMT4* (*amiR-comt4-3*, *amiR-comt4-5*, and *amiR-comt4-7*) were selected for further characterization in the T_2_ generation. Transgenic *B. distachyon* containing the empty binary vector pOL001 were used as control in all experiments. Anatomical expression data revealed that *BdCAD1* and *BdCOMT4* were highly expressed in stems; therefore, we analyzed the expression levels of the target genes in stems of the selected transgenic lines for both *BdCAD1* and *BdCOMT4* in order to confirm silencing. Quantitative real-time PCR of the *BdCAD1* or *BdCOMT4* transcript was performed to investigate the artificial microRNA induced suppression of *CAD* or *COMT*. Relative expression of *BdCAD1* was significantly reduced in *amiR-cad1-1* by 55% and *amiR-cad1-8* by 31% compared to empty vector control (Figure 5C). The expression level of *BdCOMT4* was significantly decreased by 40, 64, and 34% in lines *amiR-comt4-3*, *amiR-comt4-5*, and *amiR-comt4-7* respectively, compared to the empty vector control (Figure 5D)*.*

The velocity of CAD in crude protein extracts was determined for aboveground tissue of empty vector control and *amiR-cad1* transgenic plants as the inflorescent first emerged from the flag leaf (Additional file 1: Figure S1). Sinapaldehyde was used as a substrate to evaluate total CAD activity that includes seven other putative CAD enzymes (Additional file 1: Figure S1). Although not statistically significant, total CAD activity was reduced by 6% in *amiR-cad1-1* and 17% in *amiR-cad1-8* plants relative to empty vector control.

### Effects of downregulation of *BdCAD1* and *BdCOMT4* on development

**Figure 6:**
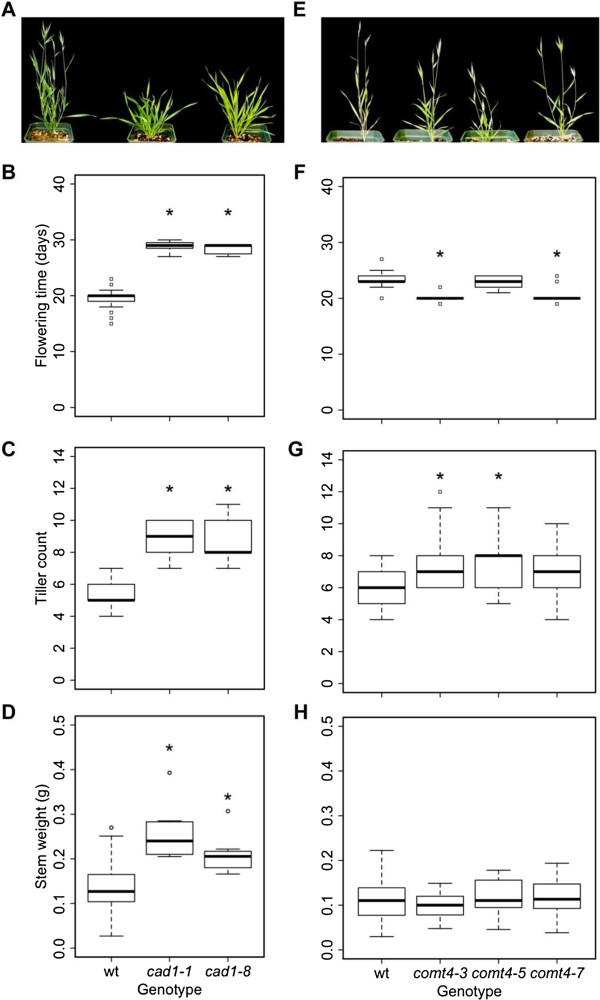
**Transgenic growth and developmental phenotypes compared to empty vector control. (A-D)***amiR-cad1*, **(E-H)***amiR-comt4*, and empty vector control. Box plots and significance are as described for Figure 5.

Transgenic plants were assayed for changes in growth and development typical of lignin deficiency including time to flower, tiller number, and stem weight. Both *CAD*-downregulated events showed a significant delay in inflorescence emergence, flowering on average ten days later than empty vector control plants (Figure 6A, B). On the other hand, *amiR-comt4-3* and *amiR-comt4-7* plants flowered significantly earlier than the empty vector control, but this difference was not observed in *amiR-comt4-5* (Figure 6E, F). The *amiR-cad1-1* and *amiR-cad1-8* events showed a significant, near two-fold increase in tiller number (Figure 6C). The tiller count among the *amiR-comt4* mutants was significantly increased relative to the empty vector control for *amiR-comt4-3* and *amiR-comt4-5* (Figure 6G). Total stem weight was measured at senescence following the removal of seeds and leaves. Events *amiR-cad1-1* and *amiR-cad1-8* showed a significant increase in stem biomass (Figure 6D), while all *amiR-comt4* events were similar to the empty vector control (Figure 6H).

### Transgenic plants have lignin-associated phenotypes

**Figure 7:**
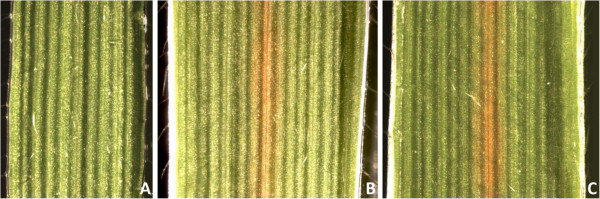
**The *amiR-cad1 *transgene results in a brown midrib phenotype.** For each line, a leaf was removed from the second node and imaged using darkfield microscopy. **(A)** empty vector control, **(B)***amiR-cad1-1*, and **(C)***amiR-cad1-8*. The brown pigmentation of the leaf midrib of transgenic *CAD*-downregulated plants was consistently observed in new leaves over time. The midrib of the empty vector control appeared green and no difference in pigmentation was observed. *COMT-*downregulated transgenics looked similar to control.

The typical brown midrib phenotype was observed in all *CAD*-downregulated plants, but not in empty vector control or *COMT*-downregulated plants (Figure 7A-C). The leaf midrib appeared a brownish tan color in leaves of *amiR-cad1-1* and *amiR-cad1-8* plants and discoloration was consistently observed in successive leaves, while the control leaves remained green until senescence. Leaf midribs in *amiR-comt4-3*, *amiR-comt4-5*, and *amiR-comt4-7* appeared similar in color to the control.

**Figure 8:**
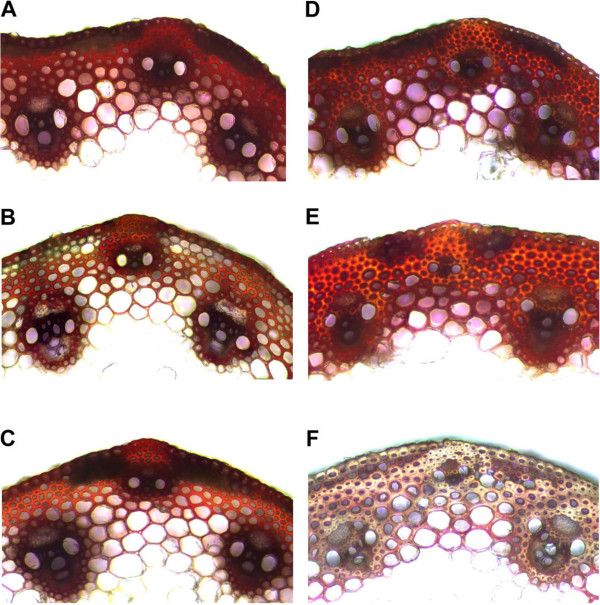
**Histochemical analysis of lignin in stem.** Cross sections of stems at the first internode of developmentally equivalent plants were dissected when the flag leaf was 4 cm below the spike. **(A)** to **(C)** Wiesner staining of empty vector control **(A)**, *amiR-cad1-1***(B)**, *amiR-comt4-5***(C)**. Wiesner reagents stain lignified tissue in a concentration-indicative manner; heavily lignified tissue stains dark red, while areas with less lignin stain orange-yellow. **(D)** to **(F)** Maule reagent staining of empty vector control **(D)**, *amiR-cad1-1***(E)**, and *amiR-comt4-5*. The Maule reagent stains S lignin; a shift from red to brown-yellow is representative of a decrease in S lignin.

The effect of changes in *BdCAD1* and *BdCOMT4* transcript abundance on lignification was evaluated by histochemical analysis. Since *CAD* is involved in the last step in the production of the precursors to S, G, and H lignin, we expected *BdCAD1*-deficient plants to be altered in lignin (Figure 1). Lignin amount and localization was observed with Wiesner staining of hand-cut stem cross sections from the first internode of developmentally equivalent transgenic and empty vector control plants. The Wiesner reaction stains lignin in a concentration-indicative manner, whereby lignified tissue stains a dark red color and less lignified tissue an orange-yellow color. Empty vector control stem stained the dark red color for lignin (Figure 8A). There was a noticeable difference in staining in both *amiR-cad1* and *amiR-comt4* stem sections relative to the control. The *amiR-cad1* stems stained a visibly lighter yellow color in regions that appeared red in the control, most notably in the sclerenchyma fibers, epidermal cells, and vascular bundle sheath (Figure 8B). The *amiR-comt4* stems stained an orange color relative to the red in the control, although this was mainly restricted to sclerenchyma fibers (Figure 8C).

Since *COMT* is important in the production of the sinapyl alcohol precursor for S lignin, we expected *BdCOMT4*-downregulated plants would exhibit a more drastic change, specifically in S lignin, more so than in total G and H lignin content (Figure 1). The Maule reagent was used to observe S lignin amount and localization in stems; the reagent stain S lignin a dark red-purple color. A shift to yellow-brown is indicative of a decrease in S lignin. Empty vector control stem sections treated with the Maule reagent stained a red-purple color (Figure 8D). Examination of the *amiR-cad1* stems revealed a reduction in red staining and a slight shift to yellow-orange color, notably in the epidermal cells and sclerenchyma fibers (Figure 8E). There was a striking decrease in staining of the *amiR-comt4* stem sections, in which the majority of tissue stained pale brown in color, with slight purple coloration seen in the outer regions of the pith (Figure 8F). The dramatic shift in color was reflective of a severe loss of S lignin in *amiR-comt4* stems compared to the empty vector control.

### Effect of *BdCAD1* or *BdCOMT4*downregulation on lignin composition

**Figure 9:**
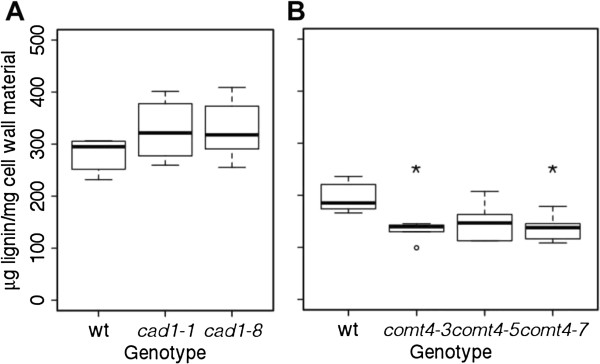
**Acetyl bromide determination of lignin content. (A)***amiR-cad1* and **(B)***amiR-comt4*. Lignin content in senesced stem was measured in transgenics and empty vector control. Box plots and significance are as described for Figure 5.

To determine total lignin polymer content, plant material was subjected to hydrolysis by acetyl bromide (AcBr) and fluorescent products, AcBr soluble lignin, were quantified by spectrometry. There was no significant difference in AcBr lignin content in *CAD*-downregulated plants as compared to the empty vector control (Figure 9A). On the other hand, AcBr lignin was significantly reduced by an average of 31.5% in *amiR-comt4-3* and *amiR-comt4-7* plants and 24% (*P* = 0.08) in *amiR-comt4-5* relative to the control.

Lignin composition was evaluated by thioacidolysis, which cleaves β-O-4 ether bonds within the lignin polymer to reveal the monomer components. The recovered monomeric degradation products, namely S, G, and H units, were quantified by gas chromatography mass spectrometry. Empty vector control samples consisted of approximately 55% S, 40% G, and 5% H lignin units. Monomer levels were altered in *amiR-cad1* and *amiR-comt4* plants (Table 1). Downregulation of *BdCAD1* was associated with a significant decrease in S units and a slight yet not statistically significant increase in G units, resulting in a reduced S/G ratio. Although H units were relatively scarce, they were increased in *amiR-cad1* plants. The *amiR-cad1-8* plants showed the most dramatic phenotype, in which the amount of S units and the S/G ratio were 16% and 22% lower than control. Although not statistically significant, the *BdCOMT4*-downregulated plants exhibited a 10% decrease in S units and a 17% reduction in G units, along with an increase in the S/G ratio relative to empty vector control plants. No consistent difference in H lignin was observed in *amiR-comt4* plants.

**Table 1:** Lignin composition in CAD1- and COMT4- downregulated transgenic plants

**Plant line**^**1**^	**S (μmol/g)**^**2,3**^	**G (μmol/g)**^**2,3**^	**H (μmol/g)**^**2,3**^	**S/G**^**2,3**^
Control 1	89.7 ± 2.3	69.5 ± 1.4	8.9 ± 0.1	1.29
*amiR-cad1-1*	87.7 ± 1.8	75.8 ± 1.8	10.0 ± 0.1	1.16
*amiR-cad1-8*	75.2 ± 2.0	75.0 ± 1.7	9.4 ± 0.2	1.00
Control 2	97.2 ± 5.6	65.7 ± 3.6	9.5 ± 0.5	1.48
*amiR-comt4-3*	86.6 ± 3.8	54.2 ± 1.4	8.9 ± 0.2	1.60
*amiR-comt4-5*	92.4 ± 1.9	60.0 ± 0.9	10.8 ± 0.2	1.54
*amiR-comt4-7*	88.5 ± 2.7	55.8 ± 1.3	9.1 ± 0.2	1.58

^1^Senesced stem tissue of *amiRcad1* and *amiRcomt4* were grown in different experiments with separate empty vector controls.

^2^Values are means ± SD (n = 3).

^3^Syringyl (S), guaiacyl (G), p-hydroxphenyl (H) units.

### Biological conversion efficiency in transgenic plants

**Figure 10:**
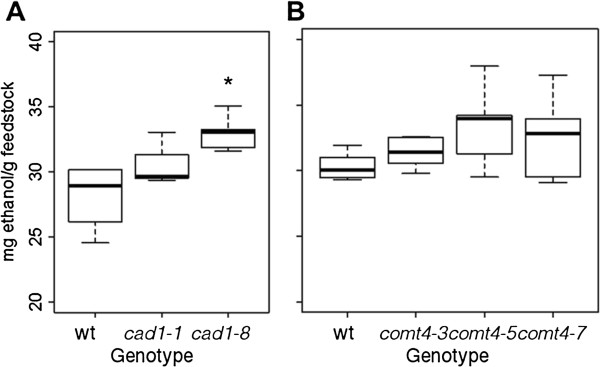
**Biological conversion efficiency with the microbial system *Clostridium phytofermentans. *(A) ***amiR-cad1 ***(B) ***amiR-comt4*. Ethanol yield from senesced stem was quantified in transgenics and empty vector control. Box plots and significance are as described for Figure 5.

In order to evaluate the impact of lignin pathway modification on biofuel feedstock quality, we measured the potential of the transgenic plants to produce ethanol following inoculation with the cell-wall-degrading, ethanogenic bacterium *C. phytofermentans*. A slight increase in average ethanol concentration was detected for both the *amiR-cad1* and *amiR-comt4* lines (Figure 10). Fermentation of the *CAD*-downregulated plants resulted in ethanol yields that were increased by 9% in *amiR-cad1-1* and 17% (*p* = 0.01) in *amiR-cad1-8* lines relative to empty vector control plants. Digestion was slightly improved by *COMT*-downregulation, as ethanol yield was increased by 4% in *amiR-comt4-3* (*p* = 0.76), 10% in *amiR-comt4-5* (*p* = 0.10), and 8% in *amiR-comt-4-7* lines (*p* = 0.24).

## Discussion

In this study, we used a candidate gene approach to identify the *CAD* and *COMT* genes involved in monolignol biosynthesis in *B. distachyon*. While well studied as key enzymes in the lignin pathway that influence forage quality, understanding of these enzymes is now of increased interest due to an apparently similar effect on biofuel feedstock quality [8]. To date, there have only been a few reports of *CAD* or *COMT* downregulation in transgenic monocots [36-39]. These species, such as *F. arundinacae* and *L. perenne* have proven to be challenging research subjects. We report here the effectiveness of artificial microRNA silencing in a species considerably more amenable to research, *B. distachyon*, from which gained knowledge can be contributed towards the optimization of bioenergy grasses.

In monocots such as *Z. mays*, *S. bicolor* and *O. sativa*, CAD tends to exist as a multi-gene family with one CAD primarily involved in monolignol biosynthesis [48,49,55]. In contrast, in the eudicot *A. thaliana*, the last step of monolignol biosynthesis is controlled synergistically by two genes, *AtCAD-C* and *AtCAD-D*[57]. Based on amino acid sequence and gene expression pattern, we identified *BdCAD1* as a likely candidate for having a role in lignin biosynthesis. Albeit with significantly lower efficiency than *BdCAD1*, Bradi4g29770 can also use coniferyl aldehyde and coniferyl alcohol as substrates and may also have a role in monolignol biosynthesis [58]. Unlike the other BdCADs, BdCAD1 contains all sequence features characteristic of a zinc-dependent alcohol dehydrogenase and more specifically, appears to be a member of the medium-chain dehydrogenase/reductase superfamily. High similarity in sequence motifs and substrate binding position suggests that BdCAD1 shares the same function as the *bona fide* CADs [56]. Consistent with multi-gene CAD families in *Z. mays*, *S. bicolor* and *O. sativa*, one particular protein in *B. distachyon* shared a significantly higher degree of homology with other known CADs than any other family member [48,49,55]. Gene expression analysis revealed that *BdCAD1* was the most highly expressed member of the gene family and that transcript abundance was particularly high in stem and root, where secondary cell walls are prevalent. Lignin-associated *CAD* expression was similarly high in stem and root tissues in *P. virgatum*, *F. arundinacae*, *O. sativa*, and *S. bicolor*[22,38,55].

The downregulation of *BdCAD1* caused phenotypes characteristic of lignin deficiency without reducing plant biomass. The delay in flowering time of the *amiR-cad1* plants is consistent with the phenotypes in five *S. bicolor bmr* mutants including *CAD* impaired *bmr6*[42,59]. The variation in flowering time observed in lignin mutants from *S. bicolor* and *B. distachyon* reinforces the possibility of an evolutionarily conserved mechanism between cell wall biosynthesis and production of flowers [60]. Because lignin has a significant role in xylem function, it is possible that changes in lignin may alter development by perturbing water transport. However, this rationale seems unlikely considering that, while *amiR-cad1* plants were developmentally delayed, mature transgenic plants were significantly larger than empty vector control plants (Figure 6D). An increase in aboveground stem biomass, even coupled with delayed flowering, is a favorable trait for a perennial energy crop, considering crop rotation will not be part of cultivation. While mutations in *CAD* can sometimes lead to pleiotropic effects of dwarfing, lodging, and a decrease in grain and/or biomass yield, these effects are mostly background-dependent [40]. Further understanding of gene-by-gene interactions that result in these deleterious effects will increase the efficiency of cultivar development.

We present here the brown midrib leaf phenotype for the first time in a C_3_ grass [40]. Genetic redundancy may explain why the phenotype has not been observed in mutant polyploid species such as *T. aestivum* and *F. arundinacae*; however, CAD mutants of diploid *O. sativa* do not exhibit a leaf brown midrib [25,55,61,62]. It has been suggested that the brown midrib phenotype may present itself differently in various species. In *O. sativa*, a mutation in *GOLD HULL AND INTERNODE 2*, which encodes a CAD enzyme, caused a red-brown pigment in the hull, internode, and basal leaf sheath while the leaf midrib did not show the same discoloration [63]. Similarly, recent research in *B. distachyon* indicated that CAD mutants displayed the red-brown pigmentation in various tissues including nodes and flowers, but not in the leaf midrib [64]. We did not observe color differences in tissues other than leaf midrib. In other species, including *Populus sp.* and *N. tabacum*, transverse stem cross-sections of transgenic *CAD*-downregulated plants exhibited unusually red xylem [13,16,20]. On the other hand, no visible mutant phenotype was observed in *CAD*-RNAi *Z. mays* plants [13]. Here, the *BdCAD1*-downregulated plants phenotypically resembled *Z. mays*, *S. bicolor*, and *P. glaucum* leaf brown-midrib mutants. The brown-midrib phenotype may occur only when CAD activity is decreased beneath a certain threshold [65]. For example, in four lines of antisense-CAD transgenic tobacco with residual CAD activity ranging from 8-56%, the extent of CAD downregulation was correlated with the presence and pattern of reddish-brown xylem [16]. We measured relatively small changes in CAD activity, but at a developmental stage that had not yet exhibited the brown-midrib phenotype. Nonetheless, the *amiR-cad1-8* plants which were most reduced in CAD activity were significantly more digestible when the plant had completely senesced. It is possible at a subsequent developmental stage characterized by greater lignin biosynthesis that diminished CAD activity would be more evident.

The quality of lignin was altered in *amiR-cad1* plants, as indicated by a significant decrease in S units observed by thioacidolysis and in agreement with histochemical staining by the Maule reagent. Previous research in *CAD*-downregulated *N. tabacum* and *CAD* mutant *B. distachyon* showed that the most dramatic change in lignin composition in plants was a severe decrease in S lignin [16,64]. Some *CAD* gene knockouts produce functional lignin through increased incorporation of cinnamyl aldehyde subunits into the lignin polymer [43]. Previous reports for *Z. mays*, *N. tabacum*, and *Populus sp.* demonstrated an incorporation of aldehydes into the lignin polymer, in which increased coniferyl aldehyde caused an increase in intensity of the Wiesner stain [13,20]. On the contrary, the Wiesner stain in *amiR-cad1* lines was less intense than control plants. A likely explanation is that the inhibition of S lignin synthesis still caused an accumulation of aldehydes, but specifically sinapyl aldehydes, which are not detected by Wiesner staining. This is consistent with decreased Wiesner staining and incorporation of 8-O-4-coupled sinapyl aldehyde in *B. distachyon* CAD mutant plants [64].

Consistent with reports in *N. tabacum*, *M. sativa*, *E. camaldulensis*, *Populus sp.*, *F. arundinacae*, and *Z. mays*, *amiR-cad1* plants were unchanged in the amount of acetyl bromide soluble lignin polymer, but thioacidolysis indicated changes in lignin monomer composition [13,16-18,20,21,24,38]. The lignin in *CAD* downregulated plants was generally more reactive. This has been illustrated by improved pulping properties in *Populus sp*., forage digestibility in *N. tabacum* and *F. arundinacae*, saccharification in *P. virgatum* and *B. distachyon*, and digestibility in *Z. mays*[13,16-18,20,22,23,25,64]. Along the same lines, the modified lignin in *amiR-cad1-8* improved biological conversion efficiency by a statistically significant 17%.

One protein among the four COMTs identified in *B. distachyon*, BdCOMT4 (Bradi3g16530), contained all of the signature features of a plant *O*-methyltransferase. Plant *O-*methyltransferases tend to have broad substrate specificity, and all nine substrate binding and positioning residues in BdCOMT4 are common to COMT proteins in other species. Similar to *L. perenne*, *F. arundinacae*, *P. tremuloides*, and *M. sativa COMT* genes [38,66-68], *BdCOMT4* was the most highly expressed COMT in stem, root, and leaf tissues than any of the three other *B. distachyon* family members. Similar to observations made in *P. tremuloides*, *BdCOMT4* expression was relatively low in leaves compared to stems [68]. Phylogeny, amino acid sequence, and the abundance of transcript in lignified tissues concurrently support that BdCOMT4 is an *O*-methyltransferase involved in monolignol biosynthesis in *B. distachyon.*

The downregulation of *COMT* resulted in changes in various phenotypic traits. Mutants tended to flower earlier than the empty vector control, as seen for the lignin mutants *bm1* in *Z. mays* and *bmr7* in *S. bicolor*[42,59]. In general, the brown midrib phenotype is not common in *COMT*-downregulated transgenic plants, with the only reports of a reddish-brown coloration of the leaf and internodes being in *Z. mays*[37,69]. Although the cause of discoloration in plants with impaired *CAD* activity is often attributed to the incorporation of aldehydes into the lignin polymer, there is no obvious correlation between the phenotype and the activities of other enzymes of the monolignol biosynthesis pathway. Previous biochemical analysis has indicated that the brown coloration is not a result of accumulated carotenoids, anthocyanins, flavones, tannins, or flavonols, but could possibly be due to incorporation of other phenolic compounds into the lignin polymer [40]. Accumulation of novel 5-OH-G units has been observed in *COMT*-downregulated transgenic *M. sativa* and *Z. mays*, although a visual phenotype associated with this phenomenon has not been defined [33,37,69,70].

In our transgenic *B. distachyon*, the perturbation of the COMT enzyme had a deleterious effect on the total quantity of lignin produced in the plant. Similarly, downregulated *COMT* mutants in *Z. mays*, *F. arundinacae*, *L. perenne*, *S. bicolor*, and *Saccharum spp.* were also reduced in total lignin [38,39,69,71]. Staining with the Maule reagent revealed an obvious difference between control and *amiR-comt4* transverse stem cross sections. We measured an increase in ethanol yield of up to 10% in *amiR-comt4* lines, which is consistent with the characterization of *COMT* mutants in other species [33,37,38,69,71-73].

## Conclusion

One of the more costly steps of producing liquid fuels from biomass on the biochemical platform is the pretreatment required to reduce biomass recalcitrance for the enzymatic and fermentation steps. As a result, there has been increasing effort to identify the factors behind biomass recalcitrance. In this study, modification of *CAD* and *COMT* expression induced changes in cell wall composition that improved amenability to conversion. A significant 17% increase in ethanol yield from plant biomass, as observed here by *CAD* downregulation, would increase industrial efficiency of processing such feedstock. Genetic modification of lignin biosynthesis may provide a means of improvement of biofuel crop conversion efficiency by reducing biomass pretreatment costs, thereby improving the bioethanol production process overall.

## Methods

### Phylogenetic analysis

Candidate CAD and COMT in *B. distachyon* were identified by amino acid homology with known proteins in other plant systems by BLAST search of the Phytozome v8.0 and NCBI databases [74,75]. Multiple amino acid sequences were aligned using ClustalW and analyzed with the associated editing program JalView 2.0 [76]. A neighbor-joining tree with bootstrap 1000 was constructed with MEGA [77]. Sequence data from this article can be found in the GenBank/EMBL databases under the following accession numbers: Sbbmr6 (BAF42789.1), Zmbm1 (ACG45271.1), PviCAD2 (ADO01602.1), OsCAD2 (NP_001046132.1), FaCAD (AAK97809.1), SoCAD (O82056.1), TaCAD (ADI59734.1), SbCOMT (AAO43609.1), Zmbm3 (NP_001106047.1), PviCOMT (ADX98508.1), TaCM (ABP63535.1).

### Microarray expression profiling

Expression patterns of the multi-gene *CAD* and *COMT* families were observed using microarray expression profiling of leaf, root, and stem tissue of *B. rachypodium distachyon.* Plants were in a growth chamber at 20°C with 20 h light:4 h dark cycles at a fluence rate of 220 μmol of photons^.^m^-2.^s^-1^ and relative humidity of 67–69. Additionally, for plate-grown plants, seeds were de-hulled and then imbibed in water for two hours with shaking. Then, seeds were treated with 70% ethanol for 20 seconds, rinsed with sterile water, then soaked in 1.3% sodium hypochlorite for 4 minutes at room temperature while shaking. Seeds were subsequently rinsed three times with sterile water and stored in the dark at 4°C for a minimum of 2 days in a sterile Petri dish with filter paper. Seedlings were grown for seven days on 0.5X Murashige and Skoog (MS) medium containing 0.7% bactoagar adjusted to a pH of 5.8 with KOH.

Approximately 30 days following germination, total leaf and stem were collected as the inflorescence emerged from the flag leaf. Leaves were collected from the stems with a curved-tip probe. Nodes and internodes from the second leaf junction to the internode below the inflorescence were placed in a tube cooled with liquid nitrogen. Seven-day-old whole seedlings were flash frozen in liquid nitrogen and then the roots were snapped off into a sterile culture tube. The six time points were collected over the course of one day at ZT2, 6, 10, 14, 18, and 22. Three plants were dissected for each time point and in triplicate for each tissue type. Samples were stored in liquid nitrogen or at −80°C until RNA extraction. Tissue was ground with mortars and pestles in liquid nitrogen. RNA was extracted using the Qiagen (Valencia, CA) Plant RNaeasy Kit according to the manufacturer’s instructions. Labeled sense strand cDNA probes were synthesized using the Ambion WT expression kit.

Transcript abundance of three biological replicates of three-week-old leaves and stems, and seven day old roots was measured using the Affymetrix *B. distachyon* BradiAR1b520742 whole genome tiling array. The array contains ~6.5 M unique 25-mer oligonucleotide features, both the forward and reverse strand sequence. The complete genome sequence is tiled with an average of 30 bases between each array feature; 1.6 million features correspond to exons and introns and 4.9 million features between gene models. Version 1.0 genome annotation includes a total of 25,532 protein coding genes and 2,542 non-coding genes [74]. Approximately ~95% (~26,670) of the genes have at least five corresponding exon array features and from those a summary value was calculated for each gene model. The average number of array features corresponding to the CAD and COMT families is 38, ranging from 10 to 111. Probeset values were calculated using gcRMA. The Affymetrix BradiAR1b520742 GeneChip data (.CEL files) have been deposited at PLEXdb [Accession no: BD3].

### Generation of transgenic plants

The WMD Version 3 web-based tool (http://wmd3.weigelworld.org) was used to design highly specific artificial microRNA (amiRNA) constructs to target the *BdCAD1* (Bradi3g06480) and *BdCOMT4* (Bradi3g16530) transcripts. This program selects a 21-mer sequence in the target gene from which an amiRNA can be produced. The gene aliases for *BdCAD1* and *BdCOMT4* were searched in the WMD-3 *B. distachyon* 1.0 genome database for the amiRNA sequence which would most likely hybridize to the target mRNA without affecting the rest of the genome. The native *BdCAD1* transcript was targeted by the amiRNA sequence AAGCGCTTACTTCTCAGATCA, corresponding to part of the fourth exon, with hybridization energy of the target site in the target gene of −38.50 kcal/mol. The native *BdCOMT4* transcript was targeted by the amiRNA sequence, ACGAAGCTCCTCGACTTATAA, corresponding to the second exon, with hybridization energy of −39.85 kcal/mol.

Modified polymerase chain reactions were performed as described in the protocols section of the WMD3 website to engineer the amiRNA sequences into the endogenous *O. sativa* microRNA precursor *osa-MIR528* in the PNW55 vector [78]. All PCRs were performed with Phusion High-Fidelity DNA Polymerase (New England Biolabs, Ipswitch, MA) according to the manufacturer’s instructions. AmiRNA precursors were then cloned into the Gateway compatible vector pENTR/D- TOPO (Invitrogen, Grand Island, NY). Following sequence confirmation, entry clones were recombined into the destination vector pOL001 using the LR Clonase II Plus enzyme (Invitrogen, Grand Island, NY). This vector confers hygromycin resistance with the *HptII* gene and will express the amiRNAs under the constitutive ubiquitin promoter *UBQ* from *Z. mays*. Electroporation was used to transform the amiRNAs into *Agrobacterium tumefaciens* strain *AGL1.*

Transgenes were integrated into the *B. distachyon* accession Bd21-3 genome by *Agrobacterium-*mediated transformation of embryogenic calli following the protocol described by Vogel and Hill [79]. Regenerated plants from *BdCAD1* transformation events *amiR-cad1-1* and *amiR-cad1-8* along with regenerated *BdCOMT4* plants from transformation events *amiR-comt4-3*, *amiR-comt4-5*, and *amiR-comt4-7* were selected for characterization in the T_2_ generation. Control plants were obtained by transformation with the empty binary vector pOL001. Plants were grown in a growth chamber at 20°C with 20 h light: 4 h dark cycles at a fluence rate of 220 μmol of photons^.^m^-2.^s^-1^ and relative humidity of 67–69.

Transgenic and empty vector control plants were genotyped by PCR of leaf genomic DNA. Leaf tissue was frozen in liquid nitrogen and pulverized with 6.35 mm stainless steel beads (BioSpec, Bartlesville, OK) in a Retsch Mixer Mill MM400. Pulverized tissue was treated with 600 μl DNA Extraction Buffer (100 mM NaCl, 50 mM Tris, 25 mM EDTA, 1% SDS, 10 mM 2-mercaptoethanol) at 65°C for 10 minutes. Samples were then placed on ice, mixed with 250 μl 5 M potassium acetate, and incubated for 20 minutes. The solution was centrifuged in a tabletop centrifuge at 12,000 rpm for 10 minutes. The supernatant was transferred to tubes containing 600 μl 100% isopropanol, mixed, and centrifuged to pellet the DNA. The supernatant was discarded and the pellet rinsed with 300 μl 70% ethanol. The pellet was resuspended in 225 μl T_10_E_5_ (10 mM Tris, 5 mM EDTA), mixed with 25 μl 3 M sodium acetate, 500 μl 100% ethanol and centrifuged in a tabletop centrifuge at 10,000 rpm for 7 minutes. Supernatant was discarded and the pellet was rinsed with 70% ethanol and centrifuged an additional 7 minutes. The pellet was allowed to air dry and was then resuspended in 30 μl T_10_E_1_ (10 mMTris, 1 mM EDTA). All putative transgenic and empty vector control plants were tested for the presence of the *HptII* transgene by PCR of leaf genomic DNA with primer forward: 5′ AGAATCTCGTGCTTTCAGCTTCGA 3′ and primer reverse: 5′ TCAAGACCAATGCGGAGCATATAC 3′. Only confirmed positive transformants were analyzed in subsequent experiments.

### Molecular characterization of transgenic plants

Transgenic and empty vector control plants were subjected to quantitative real-time PCR to assay for *BdCAD1* and *BdCOMT4* gene expression. Whole stem tissue was sampled as the inflorescence first emerged from the flag leaf, frozen in liquid nitrogen, and pulverized with 6.35 mm stainless steel beads (BioSpec, Bartlesville, OK) using Retsch Mixer Mill MM400. Total RNA was extracted from pulverized tissue using an RNeasy Plant Mini Kit (Qiagen, Valencia, CA) according to the manufacturer’s instructions; RNA purification was analyzed with the NanoDrop1000 (ThermoScientific, Waltham, MA). RNA samples were reverse-transcribed into cDNA using the SuperScript III First-Strand Synthesis System (Invitrogen, Grand Island, NY). Primers for gene-specific real-time PCR were selected using the QuantPrime design tool [80]. Reactions were completed using a QuantiFast SYBR Green PCR Kit (Qiagen, Valencia, CA) in an Eppendorf Mastercycler ep realplex2. To assay for *BdCAD1* expression, cDNA from *amiR-CAD1* and empty-vector plants was amplified with *BdCAD1*-specific primers (forward: 5′AGGATAGAATGGGCAGCATCGC 3′; reverse: 5′ ATCTTCAGGGCCTGTCTTCCTGAG 3′). To assay for *BdCOMT4* expression, cDNA from *amiR-COMT4* and empty vector plant was amplified with *BdCOMT4*-specific primers (forward: 5′ TGGAGAGCTGGTACTACCTGAAG 3′; reverse: 5′ CGACATCCCGTATGCCTTGTTG 3′). Expression values were normalized with the real-time PCR signal for the housekeeping gene Bradi5g25870 with its gene specific primers (forward: 5′- TCAGCAGGGTGCTAATTCAGTTC 3′; reverse: 5′ CGACAGAGTTTAGCGGTCTTAGC 3′). The selected housekeeping gene Bradi5g25870 exhibits moderate expression levels and extremely low variance across numerous *B. distachyon* array experiments. All qRT-PCR reactions were performed in triplicates.

### Enzymatic assays

CAD activity was determined by generally following previously published procedures [50,81-83]. Approximately 150 mg of frozen plant tissue that had been ground as previously described and stored at −80°C was taken up in 700 μL buffer (100 mMTris-Cl, pH 7.5, 5 mM DTT, 5% ethylene glycol) and then sonicated to disrupt cell walls using a sonicator equipped with a micro-tip (Branson Digital Sonifier 450, Branson Ultrasonic Corp., Danbury CT). Sonicated extracts were centrifuged at 4°C for 10 min. at 14,000 RPM and the crude protein extract was placed into new 1.5 mL tubes and kept on ice. Each plant sample was extracted twice and enzyme activity was tested by monitoring absorbance changes on a microplate reader (BioTek Synergy HT, BioTek Instruments, Winooski, VT) at A_340_. All reactions were carried out in a volume of 200 μL and consisted of 100 mM MES at pH 6.5, 200 μM NADPH, 100 mMsinapyl aldehyde, and 10 μL of the crude protein extract. Absorbance changes were monitored for three minutes after addition of crude protein extract. Enzyme velocities were determined by fitting a line using linear least squares to the absorbance data; the slope of the line was used in velocity determinations. Protein concentrations were determined using the Pierce 660 nm protein assay (Pierce Biotechnology, Rockford, IL) and served to normalize the calculated velocity for each extract.

### Histochemical staining of lignin

Cross sections of stems were manually dissected from the first internode of developmentally equivalent transgenic and control plants. The Wiesner staining method [84] was used to visualize total lignin content and localization in the stem. Sections were stained with 1% phloroglucinol for 2 minutes followed by a wash in 50% HCl and were mounted onto microscope slides for observation. The Maule reagent [85] was used to observe S lignin content and localization in stems. Sections were treated with 1% KMnO_4_ for 5 minutes and rinsed with water. Sections were then treated with 10% HCl for 2 minutes, rinsed with water, and mounted on microscope slides in 1% NH_4_OH. Images were captured using a Nikon Eclipse E200MV R microscope with a 3 PixeLINK 3 MP camera.

### Lignin content and composition analysis

Senesced stem material was dried thoroughly following wash steps with 70% ethanol at 65°C for 1 hour, and following ethanol removal was rinsed with acetic acid and allowed to air dry. Stem was pulvarized into powder with metal beads in a Retsch Mixer Mill MM400. Acetyl-bromide-soluble lignin was quantified using the procedure described by Foster et al. [86]. Triplicate samples of 1.5 mg powdered stem material were used for AcBr lignin analysis. Lignin composition was evaluated by thioacidolysis and S, G, and H unit quantification was conducted as described by Foster et al. [86].

### Digestibility analysis

Biological conversion efficiency was measured using the microbial system developed for *Clostridium phytofermentans*, which converts plant biomass to ethanol, following the protocol as described by Lee et al. [72]. Triplicate 20 mg samples, dried and prepared as described above, were incubated with *C. phytofermentans* in 96-well plates and supernatant ethanol concentration was measured with high-performance liquid chromatography (HPLC) with RI detection [87].

### Statistical analysis

For each measurement, 4 to 17 independent plants were sampled. Analysis of variance and Dunnett’s contrasts were performed in R v2.15.0.

## Competing interests

The authors declared that they have no competing interests.

## Authors’ contributions

GMT and SPH designed research; GMT, DAM, SJL, and AJS performed research; HDP, TCM, GS and SPH contributed new reagents/analytic tools; SPH and HDP analyzed data; and GMT and SPH wrote the paper. All authors read and approved the final manuscript.

## Supplementary Material

Additional file 1: Figure S1 CAD enzyme activity in empty vector control and *amiR-cad1* transgenic plants. Activity of CAD was measured in aboveground tissue using sinapaldehyde as a substrate. Box plots and significance are as described for Figure 5. Click here for file
