# Supplementary figures and images for: Functional characterization of cinnamyl alcohol dehydrogenase and caffeic acid O-methyltransferase in Brachypodium distachyon
Source: BMC Biotechnol. 2013 Jul 31;13:61. doi: 10.1186/1472-6750-13-61 (PMC3734214; doi:10.1186/1472-6750-13-61)

## SUPPLEMENTAL FIGURE 1

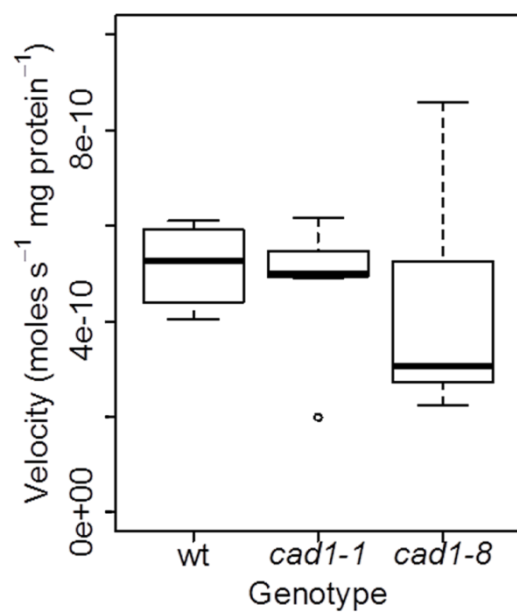

Supplement: Additional file 1: Figure S1 — CAD enzyme activity in empty vector control and amiR-cad1 transgenic plants. Activity of CAD was measured in aboveground tissue using sinapaldehyde as a substrate. Box plots and significance are as described for Figure 5. [file 1472-6750-13-61-S1.pdf]
